# Excitatory Repetitive Transcranial Magnetic Stimulation Over Prefrontal Cortex in a Guinea Pig Model Ameliorates Tinnitus

**DOI:** 10.3389/fnins.2021.693935

**Published:** 2021-07-22

**Authors:** Jack W. Zimdahl, Harrison Thomas, Samuel J. Bolland, Kerry Leggett, Kristin M. Barry, Jennifer Rodger, Wilhelmina H. A. M. Mulders

**Affiliations:** ^1^School of Human Sciences, University of Western Australia, Crawley, WA, Australia; ^2^School of Biological Sciences, University of Western Australia, Crawley, WA, Australia; ^3^Perron Institute for Neurological and Translational Research, Crawley, WA, Australia

**Keywords:** repetitive transcranial magnetic stimulation, tinnitus, guinea pig, prefrontal cortex, hyperactivity, calcium-binding protein, medial geniculate nucleus

## Abstract

Tinnitus, a phantom auditory perception that can seriously affect quality of life, is generally triggered by cochlear trauma and associated with aberrant activity throughout the auditory pathways, often referred to as hyperactivity. Studies suggest that non-auditory structures, such as prefrontal cortex (PFC), may be involved in tinnitus generation, by affecting sensory gating in auditory thalamus, allowing hyperactivity to reach the cortex and lead to perception. Indeed, human studies have shown that repetitive transcranial magnetic stimulation (rTMS) of PFC can alleviate tinnitus. The current study investigated whether this therapeutic effect is achieved through inhibition of thalamic hyperactivity, comparing effects of two common clinical rTMS protocols with sham treatment, in a guinea pig tinnitus model. Animals underwent acoustic trauma and once tinnitus developed were treated with either intermittent theta burst stimulation (iTBS), 20 Hz rTMS, or sham rTMS (10 days, 10 min/day; weekdays only). Tinnitus was reassessed and extracellular recordings of spontaneous tonic and burst firing rates in auditory thalamus made. To verify effects in PFC, densities of neurons positive for calcium-binding proteins, calbindin and parvalbumin, were investigated using immunohistochemistry. Both rTMS protocols significantly reduced tinnitus compared to sham. However, spontaneous tonic firing decreased following 20 Hz stimulation and increased following iTBS in auditory thalamus. Burst rate was significantly different between 20 Hz and iTBS stimulation, and burst duration was increased only after 20 Hz treatment. Density of calbindin, but not parvalbumin positive neurons, was significantly increased in the most dorsal region of PFC indicating that rTMS directly affected PFC. Our results support the involvement of PFC in tinnitus modulation, and the therapeutic benefit of rTMS on PFC in treating tinnitus, but indicate this is not achieved solely by suppression of thalamic hyperactivity.

## Introduction

Tinnitus is a phantom auditory percept, often described as a ringing or buzzing in the ear, which affects 10–15% of the population (Baguley et al., 2013). For about 1.5% of sufferers tinnitus severely affects quality of life, leading to loss of sleep and concentration, anxiety, stress, and suicidal ideation (Baguley et al., 2013). The precise neural substrate is still debated (Baguley et al., 2013), and different mechanisms may exist for subtypes of tinnitus (Vanneste et al., 2019), both factors contributing to the lack of a current universal cure. Tinnitus is commonly associated with cochlear damage and some degree of hearing loss. This leads to changes in central auditory structures (Tan et al., 2013), such as increased neural synchrony, and increased spontaneous and bursting activity (Norena and Eggermont, 2003; Mulders and Robertson, 2009; Kalappa et al., 2014; Basura et al., 2015; Wu et al., 2016), which may all contribute to tinnitus generation (Eggermont and Roberts, 2015).

Alongside its complex ascending projections, the auditory system also encompasses an elaborate system of descending pathways (Schofield, 2011). These descending pathways allow for modulation of the auditory information *en route* to cortex and are therefore in a prime position to affect auditory perception (see for example: Guo et al., 2017; Homma et al., 2017; GuinanJr., 2018). In addition, efferent modulation of ascending auditory information also arises from non-auditory brain structures involved in emotional and memory processing such as the prefrontal cortex (PFC), amygdala and hippocampus (Scannell et al., 1999; Rolls, 2015), further contributing to the conscious perception of auditory information (McCormick, 1992). More specifically, it is proposed that these non-auditory inputs play an important role in sensory gating, inhibiting non-salient signals (Rauschecker et al., 2010; De Ridder et al., 2014). This has now led to the hypothesis that failure of gating for non-salient signals may be involved in the generation of tinnitus (Rauschecker et al., 2010, 2015; De Ridder et al., 2014).

Emerging data suggest that tinnitus may be due to the breakdown of sensory gating circuitry between the auditory system and non-auditory limbic regions (Rauschecker et al., 2010; De Ridder et al., 2014), such that altered activity due to cochlear damage reaches the cortex and ultimately leads to a conscious percept (Rauschecker et al., 2010, 2015; De Ridder et al., 2014). Specifically it has been suggested when the increased spontaneous activity in tinnitus patients fails to be ignored and is recognized as sound, this leads to a resetting of auditory predictions allowing the tinnitus to continue (Sedley et al., 2016, 2019). Debate does exist to the cause of the increased spontaneous activity as some suggest an increased neural gain (Sheppard et al., 2020) whereas others argue the increased neural gain is underlying hyperacusis but not tinnitus (Brotherton et al., 2015).

Part of the proposed circuitry involved in sensory gating involves indirect pathways from ventromedial PFC and/or anterior cingulate cortex to auditory thalamus (medial geniculate nucleus [MGN]) (O’Donnell et al., 1997; Anderson et al., 2006; Zikopoulos and Barbas, 2006; Yu et al., 2009; Crippa et al., 2010; Smith et al., 2012; Bartlett, 2013; Mellott et al., 2014; KeiferJr., Gutman et al., 2015; Rauschecker et al., 2015). Indeed, we have shown in rats that neural activity in MGN can be modulated by input from nucleus accumbens and PFC (Barry et al., 2015, 2017). Rodent PFC as targeted in our studies is thought to be analogous to ventromedial PFC and anterior cingulate in humans (Bicks et al., 2015; Laubach et al., 2018).

Clinical studies in tinnitus patients have shown that non-invasive stimulation of PFC, using techniques such as repetitive transcranial stimulation (rTMS) and direct current stimulation, can alleviate tinnitus loudness and distress (Kleinjung et al., 2008; Vanneste et al., 2011; De Ridder et al., 2013; Lehner et al., 2013; Langguth et al., 2014). Indeed this was recently replicated in a randomised placebo-controlled, single-blinded clinical trial using high frequency rTMS over the dorsomedial PFC, which showed a statistically significant reduction in tinnitus severity (Ciminelli et al., 2020). However, these human studies cannot establish whether these beneficial effects are due to inhibition of thalamic hyperactivity as suggested by the dysfunctional gating theory (Rauschecker et al., 2010; De Ridder et al., 2014). In the present study, we implemented our guinea pig model of acoustic trauma (AT) and tinnitus (Mulders et al., 2014a, 2016) to investigate the effects of two excitatory rTMS protocols, 20 Hz and intermittent theta burst stimulation (iTBS) applied over PFC, and assessed neuronal firing patterns in MGN as well as behavioral signs of tinnitus. The MGN was selected as a target to measure neuronal activity as we have previously shown that it shows increased spontaneous firing rates in the presence of tinnitus (Cook et al., 2021) and electrical stimulation of PFC evokes changes in MGN neuronal firing patterns (Barry et al., 2017). Modeling of the rTMS coil was used to assess the extent and strength of the electric fields (E-fields) and to investigate whether the MGN could be directly activated. Excitation of PFC was hypothesized to have inhibitory effects on thalamic and therefore cortical activity, and in this way, attenuate tinnitus. In addition, we investigated the effects of rTMS on PFC, through quantitative immunohistochemical analysis of calcium binding proteins, as studies have shown changes in these proteins following rTMS treatment (Benali et al., 2011; Hoppenrath and Funke, 2013; Volz et al., 2013; Labedi et al., 2014; Mix et al., 2014; Mulders et al., 2019).

## Experimental Procedures

### Animals and Experimental Design

All experiments were in line with the Code of the National Health and Medical Research Council of Australia and the National Institutes of Health Guide for the Care and Use of Laboratory Animals (NIH Publications No. 80-23, revised 1996) with approval from the Animal Ethics Committee of The University of Western Australia (RA/3/100/1458). All efforts were made to minimise the number of animals used and their suffering. Twenty-seven pigmented adult guinea pigs of either sex (20 males; 7 females) were used. One additional guinea pig (female 875 g) was used for MRI imaging.

Detailed descriptions of behavioral testing for tinnitus (Robertson et al., 2013; Mulders et al., 2014a, 2016; Leggett et al., 2018); AT surgery (Mulders and Robertson, 2009; Mulders et al., 2011, 2014a, b; Mulders and Robertson, 2011; Vogler et al., 2011; Robertson et al., 2013); electrophysiological recordings in MGN including burst firing analysis (Barry et al., 2019; Mulders et al., 2019); and immunohistochemistry with associated quantitative analysis to determine densities of calcium-binding neurons in PFC (Mulders et al., 2019) have been described in previous papers from our laboratory and will be described only in brief in the next sections.

### Behavioral Tests for Tinnitus

Behavioral testing for tinnitus consisted of gap prepulse inhibition of acoustic startle (GPIAS) in combination with prepulse inhibition (PPI). Tinnitus testing was performed before (to obtain baseline measures) and after AT for all animals. There was at least 1 day between testing sessions and a maximum of three testing days per week to limit habituation effects. During behavioral testing, animals were lightly restrained in clear polycarbonate holders and placed on force transducing platforms in a soundproof room. Animals were left to acclimatize for 5 min prior to each testing session. Each animal was allocated to the same platform for all subsequent testing.

Both GPIAS and PPI involve the delivery of a startle tone after which the amplitude of the animals’ startle response is recorded. The startle stimulus was identical in both PPI and GPIAS conditions (1 kHz, 0.5k Hz bandwidth; 106 dB SPL; 50 ms duration) and was delivered by a speaker positioned approximately 5 cm above the animals’ head (Radio Shack 401278B). An additional speaker (Beyer DT 48) was positioned approximately 3 cm above the animals’ head to deliver the continuous background noise (GPIAS), or prepulse stimulus (PPI).

Prepulse inhibition occurs when a weak prepulse stimulus inhibits the startle response to a succeeding stronger stimulus. The PPI sessions consisted of 50 trials. In half of the trials, a 50 ms prepulse was presented 100 ms before the delivery of the startle tone, in the other half, no prepulse was present. The prepulse was a narrowband noise centered at either 8 kHz (10 dB bandwidth 2.2 kHz) or 14 kHz (10 dB bandwidth 1.6 kHz) at 66 dB SPL intensity. These two center frequencies were selected as they are either below the AT frequency in a compound action potential (CAP) audiogram region not showing threshold loss or just above the AT frequency in a CAP audiogram region showing significant threshold loss. The interval between startle stimulus presentations varied randomly by 20–30 s, and the order of the prepulse and non-prepulse trials was randomized.

Gap prepulse inhibition of the acoustic startle testing is a variant of PPI in which a silent gap, functioning as a prepulse, is inserted in a continuous background noise preceding the startle tone. GPIAS also consisted of 50 trials, with half of the trials incorporating a gap. The background noise was at the same level and characteristics as the pre-pulses in the PPI test. During testing, the animals’ startle is measured from a startle platform output and is calculated as root mean square (RMS) of force produced during the baseline response (before startle) and startle response.

For analysis, the startle response of prepulse trials was compared with no prepulse trials (for PPI) or gap trials with no gap trials (for GPIAS) within each animal. Outliers in trials were identified through RMS values ± 3 SD from the mean. Mean PPI or GPIAS suppression was expressed in percentage by comparing the RMS force between the prepulse and no prepulse trials or between the gap and no gap trials, respectively. An animal is considered to “pass” if there is a significant difference (Mann–Whitney test, *p* < 0.05) between prepulse/no prepulse or gap/no gap trials, and “fail” if the condition is not met (Mann–Whitney test, *p* > 0.05). All animals passed the PPI test once and the GPIAS paradigm twice indicating stable baselines before AT.

Possible tinnitus development was assessed after AT surgery via weekly GPIAS testing. Animals may fail GPIAS due to tinnitus but also alternatively because of either hearing loss or deficits in the neural circuitry underlying startle response and PPI and hence animals were required to pass PPI after AT to ensure that failure of GPIAS was specifically related to tinnitus. Animals that failed GPIAS on two repeat occasions (at least 1 day between sessions) and passed PPI, were considered to have behavioral signs of tinnitus. For group comparisons, GPIAS suppression was averaged over two sessions before the AT, at the time of tinnitus development and after sham or active TMS treatment preceding the final electrophysiological experiment.

### Acoustic Trauma Surgery

Acoustic trauma surgery consisted of opening the tympanic bulla to enable placement of a recording electrode on the round window of the cochlea and the measurement of thresholds of the auditory nerve before and after an AT. For anesthesia, animals received a subcutaneous injection of atropine 0.1 ml (Atropine, atropine sulfate 0.6 mg/ml, Apex Laboratories, Somersby, Australia) followed by 5 mg/kg Diazepam (Pamlin, diazepam 5 mg/ml, Ceva Animal Health, Glenorie, Australia) intraperitoneally, an intramuscular injection of 1 ml/kg Hypnorm (Hypnorm, 0.315 mg/ml fentanyl citrate and 10 mg/ml fluanisone, VetaPharma, Leeds, United Kingdom), and a subcutaneous injection of 0.1 ml lignocaine (Lignocaine20, 20 mg/ml lignocaine HCl, Troy Laboratories, Glendenning, Australia). Once surgical anesthesia was obtained, animals were placed on a heated platform in a soundproof room and mounted in hollow ear bars. Anesthesia level was maintained throughout surgery with an additional administration (one-third of the initial dose) of Hypnorm.

To assess hearing, a small incision and small hole in the tympanic bulla allowed for an insulated silver wire to be placed onto the round window. CAP thresholds were measured in response to pure tone stimuli (10 ms duration, 4/s, frequency range: 4–24 kHz) created in a closed sound system using a 1/2 inch condenser microphone driven in reverse (Bruel and Kjaer, type 4134). CAP signals were recorded using a custom-made computer program (sample rate 96 kHz, Neurosound; MI Lloyd). CAP signals were amplified (1000x), filtered (100 Hz – 3 kHz bandpass) and recorded (Powerlab 4SP, AD instruments). Then a unilateral AT (left ear, 10 kHz, 124 dB SPL, 2 h) was performed, whilst the contralateral ear (right ear) was blocked with plasticine. After AT, another CAP audiogram was measured, the incision was sutured, and animals were allowed to recover.

### rTMS Protocols

rTMS was delivered using a commercially available animal-specific coil (Cool-40 Rat Coil; Magventure, Farum, Denmark). Machine stimulus output was set to 23% to avoid direct facial muscle twitching (assessed visually). Treatment was performed on awake animals whilst being gently held in the experimenter’s lap for 10 min daily, on weekdays only, over a 2-week period. The peak induced electric field was marked allowing for positioning of the coil to be placed against the animals head over the PFC 14.2 mm anterior of the interaural line (Rapisarda and Bacchelli, 1977). This was achieved through shaving the animals’ head and marking the position with a permanent marker using handheld electronic calipers measuring from the interaural line; the interaural line was judged using stretched tape between the center of the ear canals.

Two excitatory stimulatory rTMS protocols that were based on clinical settings (Huang et al., 2005; Kleinjung et al., 2008; Lehner et al., 2013; Langguth et al., 2014) and that were similar in duration and pulse number, were used. The first, 20 Hz (2000 pulses in 10 min stimulation) was applied in 40 blocks of 50 pulses with 13 s intervals to avoid coil overheating. The other protocol, iTBS, included 2 s of stimulation trains, 10 pulses of three bursts each at 50 Hz, with an 8 s inter-train interval (1800 pulses over a 10 min period). Sham stimulation was delivered at 20 Hz, but with the coil positioned 20 cm above the animals’ head, and angled perpendicular to the animals’ longitudinal axis. The sham rTMS procedure generated the same sound as verum rTMS, but gauss meter recordings at the same position as the animals’ heads produced no detectable magnetic field. At the same time a cardboard sham coil with similar dimensions and color was held on the head to simulate coil placement sensation.

### rTMS E-Field Modelling

**FIGURE 1:**
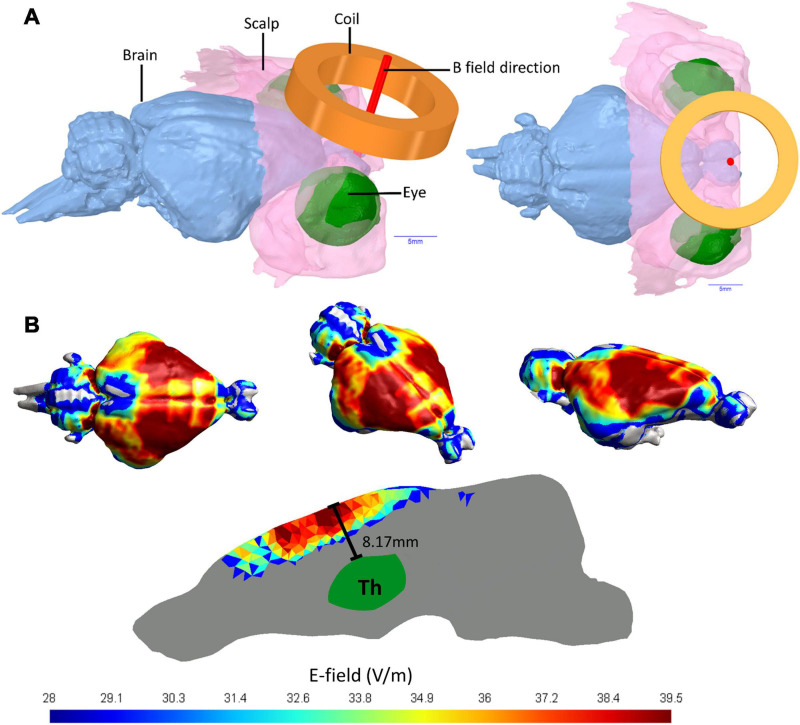
Guinea pig head 3D model and E-fields in guinea pig brain. **(A)** MRI imaging of a Guinea pig head utilized to produce a 3D head model showing the brain, the coil position held 2 mm from the scalp and the E-field direction of the active coil pointing directly into the brain. **(B)** The E-fields in V/m for simulated TMS stimulation of a guinea pig brain using a MagVenture Cool-40 Rat coil at 23% of maximum stimulator output.

The rTMS based E-fields induced in guinea pig brains in this current study were simulated using 3D computer models to investigate the E-field spread and intensity. The 3D head model was produced from anatomical T2 weighted MRI images with 100 μm thick isotropic voxels (336 slices) of a guinea pig head, which were attained using a 9.4T Bruker Biospec 94/30 small animal MRI machine. The head model was segmented into different tissue types to account for differences in conductivity. The brain was segmented out using FMRIB software library (Jenkinson et al., 2012) via the function Brain Extraction Tool (BET), while the non-brain tissues were segmented out using the program ITK-SNAP (Yushkevich et al., 2016) based on MRI image intensity values. ITK-SNAP was used to produce 3D surfaces and the software package ANSYS Academic (SpaceClaim 2019 R3, Release 19.5.0) was used to build the 3D mesh with further optimization through MeshFix (Attene, 2010). The triangle and tetrahedral based 3D FEM brain model was produced in Gmsh and the resulting model contains ∼7,152,380 tetrahedral elements and ∼1,290,940 triangles. The whole brain volume is 2.76 cm^3^ and Coil distance was set at 2 mm above the scalp to account for fur (Figure 1A). Coil definition files were provided by the program SimNIBS 3.1.2 (Saturnino et al., 2019), which was also used to solve the finite element method based rTMS simulation. The Cool-40 Rat coil file analysis, model processing and SimNIBS output analysis were conducted in MATLAB (R2016a & R2017a, The Mathworks, Inc., Natick, MA, United States). SimNIBS uses GetDP solver (Geuzaine, 2007) to calculate simulated rTMS induced E-field values. Default isotropic conductivities were set for the soft tissues (σ = 0.465 S/m), skull (σ = 0.01 S/m), CSF (σ = 1.654 S/m), gray matter (σ = 0.275 S/m), and white matter (σ = 0.126 S/m) (Wagner et al., 2004). E-field distributions were calculated with the Cool-40 Rat coil positioned tangentially to scalp surface and the stimulator output was set to 23% of maximum stimulator output as per the rTMS protocols. E-field spread over the brain tissue would be the same for the iTBS and 20 Hz protocol.

### Electrophysiological Recordings and Burst Firing Analysis

Three days after cessation of the sham or active rTMS treatments, a final GPIAS test was performed and followed by an electrophysiological experiment using extracellular recordings to assess MGN neuronal activity. During this non-recovery surgery animals received a 0.1 ml subcutaneous injection of atropine and an intraperitoneal injection of 30 mg/kg pentobarbitone sodium (Pentobarbitone, pentobarbitone sodium 60 mg/ml, Troy Laboratories, Glendenning, Australia), followed by a 0.15 ml (fixed dose) intramuscular injection of Hypnorm. To maintain anesthesia 0.15 ml of Hypnorm was given hourly and half doses of pentobarbitone sodium were given every 2 h. A tracheotomy was performed, and animals artificially ventilated on carbogen (95% O_2_, 5% CO_2_). Animals were placed on a heated platform in a soundproof room and mounted in hollow ear bars. An electrocardiogram (ECG) was recorded throughout surgery to ensure depth of anesthesia and monitor physiological status. ECG was expressed by measurements of the interval between QRS complexes (ECG interval) which were continuously displayed during the experiment on an oscilloscope. In a previous experiment (Cook et al., 2021) we have demonstrated that spontaneous firing rates in MGN significantly decrease with ECG intervals > 300 ms. Therefore, recordings were only included and analyzed while the ECG intervals were under 300 ms. At the conclusion of the experiment, animals were euthanized via an injection of fixed dose of 0.3 ml Lethabarb (sodium pentobarbitone 325 mg/ml; Virbac, Milperra, Australia).

Additionally, during this non-recovery surgery bilateral CAP audiograms were measured using the method detailed above (AT surgery). Following CAP audiograms, a partial craniotomy was performed over the cortex overlying the right MGN, contralateral to the AT exposed ear (Rapisarda and Bacchelli, 1977). The craniotomy was covered with 5% agar in saline to improve stability of recordings and prevent dehydration of the neural tissue. The contralateral right ear was blocked with plasticine. A glass insulated tungsten microelectrode was advanced along the dorso-ventral axis through the cortex to the MGN. Entry into MGN was indicated by noise-evoked cluster activity, approximately 6–7 mm from the cortical surface. Single neuron characteristic frequency (CF) and threshold were determined audio-visually using the Neurosound software when possible, and the spontaneous firing rate of the neurons was measured during a 10 s period. Immediately after these measurements, 2 min of spontaneous firing rate were recorded on LabChart (ADInstruments) in the absence of sound, to allow for post-experimental analysis of burst firing. Single neuron recordings continued until at least 50 neurons were recorded per animal or until the ECG intervals exceeded 300 ms.

Recording location in MGN was confirmed via histological analysis of electrode tracks (data not shown). In each animal 4–8 tracks were required to collect sufficient recordings. Onset latencies varied considerably from 12 to >200 ms. Neuronal CF could not always be determined as some neurons responded only to broadband stimuli. In addition, a very small number of neurons could not be driven by sound, but since they were found in between other neurons that did show a robust response to sound, they were still included in analysis. No attempt was made to identify the subdivision of the MGN. Subdivision identification would have required more recording time per neuron, and this was not compatible with our goal to collect data from 50 neurons per animal whilst maintaining the required physiological status.

### Burst Firing Analysis

Individual neurons were isolated based on amplitude and wave shape from the 2-min neuronal recordings, using the LabChart spike histogram software (ADInstruments). The individual neuron data were imported into NeuroExplorer v.4.135 (2014) software. Analysis of burst firing was performed using the burst analysis function which measured bursts per minute, and mean burst duration (Kepecs and Lisman, 2004; Kalappa et al., 2014; Kimura and Imbe, 2015). Burst firing criteria were selected based on previous studies and on visual inspection of the bursting patterns observed on the LabChart (ADInstruments) files and were set as follows: maximum interval to start burst (8 ms), maximum interval to end burst (8 ms), minimum interval between bursts (15 ms), minimum duration of burst (6 ms), and minimum number of spikes per burst (3). Neurons were only included if they were clearly distinguishable from other neurons in the recording and spike height was constant throughout the 2 min of recording. In addition, only neurons with a spontaneous firing rate > 1 spike/sec were used for subsequent burst firing analysis.

### Histology and Immunohistochemistry

Following electrophysiological recordings, animals were euthanized (0.4 ml intraperitoneal injection of Lethabarb – sodium pentobarbitone 325 mg/ml; Virbac, Milperra, Australia), and transcardially perfused with saline followed by 4% paraformaldehyde in 0.1 M phosphate buffer. Brains were removed and stored in fixative overnight followed by 30% sucrose in 0.1 M phosphate buffer solution for 48 h at 4^*o*^C. Sections were cut at 60 μm using a freezing microtome, mounted on gelatine coated slides, stained with Toluidine Blue and coverslipped. Electrode tracts were photographed using light microscopy (Nikon Eclipse 80i) connected to a camera (DigiSight) using NIS Elements Advanced Research software (Nikon).

Immunohistochemistry was performed on two series of the PFC, staining every 1 in 7 free-floating sections to investigate calcium-binding proteins (calbindin and parvalbumin). Primary antibodies were mouse-anti-parvalbumin (1:500, Sigma-Aldrich, MO, United States), and mouse-anti-calbindin (1:500, Sigma-Aldrich, Missouri, United States) and secondary antibody donkey-anti-mouse (1:500, Merck KGaA, Darmstadt, Germany). Sections were incubated overnight at 4^*o*^C in blocking solution (0.1 M phosphate buffer; 0.1% BSA; 0.3% Triton; 5% donkey serum) containing the primary antibody. Following overnight incubation, sections were incubated at room temperature for 90 min in blocking solution with the secondary antibody followed incubation in avidin-biotin complex (1:800 A and B in 0.1 M phosphate buffer; 90 min at room temperature). Staining was visualized using 3,3’-diaminobenzidine as a chromogen. Finally, sections were mounted on gelatine coated slides, dehydrated, cleared in xylene, and coverslipped with Entellan (Merck KGaA, Darmstadt, Germany).

Immunolabeled neurons were counted in both hemispheres within each mounted cortical section using imaging software (NIS-elements software). Four regions of interest (ROI) were investigated, including two dorsal regions and two ventral regions (see section “Results” for location). The ROI were selected based on distance away from the skull, and hence the overlying coil (dorsal ROI 1 and 2 vs. ventral ROI 3 and 4) and as representing more superficial (ROI 1 and 4) vs. deeper layers of the cortex (ROI 2 and 3), though we did not attempt to only capture particular layers. The areas were selected based on landmarks which enabled the same criteria to be applied in each section and ensured avoidance of double counting. The counting area was set to 0.24 mm^2^ using a 20X objective lens, with images captured using a Nikon Eclipse 80i and photomicrographs taken with an integrated Digital Sight Camera (NIS-elements software). Immunostained neurons were only counted when soma and at least two dendrites were visible.

### Statistical Analysis

Statistical analysis was performed using GraphPad Prism. Normality was checked with Shapiro–Wilks normality test. RMS startle response data was compared using a Mann–Whitney test. Group behavioral data (GPIAS), immunohistochemistry, and CAP thresholds were analyzed using two-way repeated measures analyses of variance (ANOVA) with associated Turkey’s *post hoc* tests. Spontaneous and burst firing frequency between regions were analyzed using Kruskal–Wallis test with Dunn’s multiple comparisons as the data were non-parametric.

## Results

**FIGURE 2:**
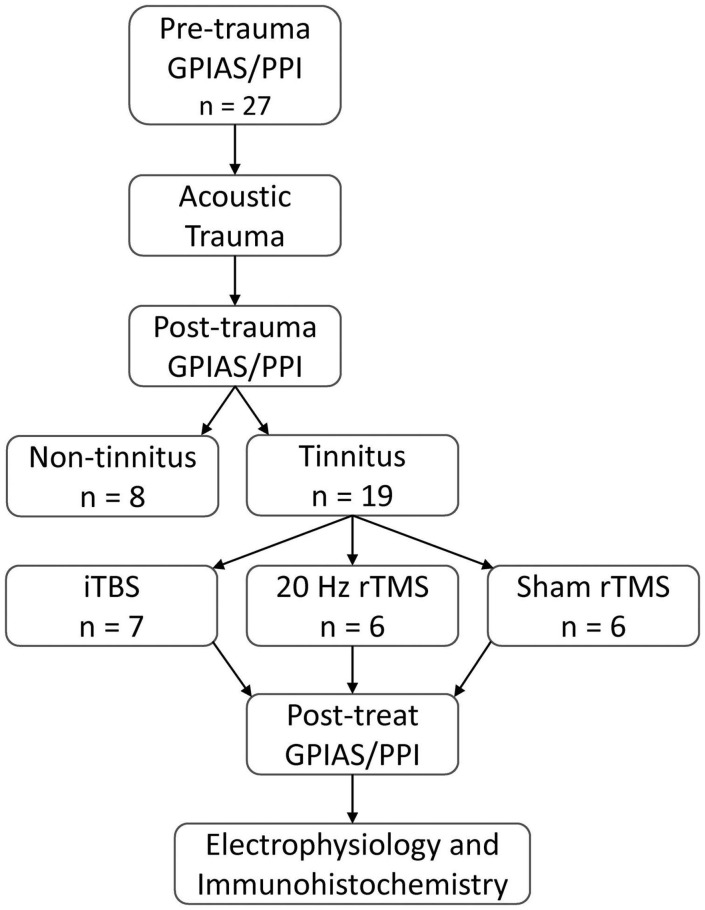
Experimental overview. All animals tested for baseline GPIAS and PPI ensuring no pre-existing behavioral evidence of tinnitus and normal startle circuitry. Baseline behavioral tests were followed by an AT to induce hearing loss. Following hearing loss, animals were tested weekly for behavioural signs of tinnitus. Tinnitus animals were treated with active rTMS (20 Hz or iTBS) or sham rTMS administered over the PFC for 10 min daily, over 2 weeks (Monday to Friday). Following treatment, behavioral signs of tinnitus were assessed, along with spontaneous and burst firing rates of single neurons in the MGN, and subsequent post-euthanasia immunohistochemical analysis of calcium-binding proteins in the PFC.

The present study aimed to compare the effects of two common clinical rTMS protocols with sham treatment applied on PFC, in a guinea pig tinnitus model. The experimental design is shown in Figure 2. Baseline behavioral tinnitus testing was performed to establish that the neural circuitry underlying startle response and PPI was normal and to ensure there was no evidence of pre-existing tinnitus. Then surgery was performed to allow for measurement of auditory thresholds and to allow for exposure to a unilateral AT which caused permanent hearing loss. After a 1-week recovery period from surgery, animals resumed weekly behavioral testing for tinnitus. Once animals presented with behavioral signs of tinnitus, active rTMS (either 20 Hz or iTBS) or sham rTMS was administered over the PFC of the awake animal for 10 min, weekdays only, over a period of 2 weeks. Three days following cessation of this treatment, behavioral signs of tinnitus were reassessed, after which spontaneous firing of single neurons in the MGN was recorded under deep anesthesia. Brain tissue was subsequently obtained for immunohistochemical analysis of calcium-binding proteins in PFC.

### Electromagnetic Field Modelling

Using 9.4 T MRI for 3D head imaging, these computer models were then used to investigate E-field spread and intensity (Figure 1). The 99th percentile highest E-field value produced in the cortex with the Cool-40 Rat coil set to 23% machine stimulus output was 39.5 V/m, 0.457 cm^3^ of this brain volume contained an E-field of 36.23 V/m or above and 1.75 cm^3^ measured an E-field of 24.15 V/m or above. The maximum E-field value found in the thalamus was 20.4 V/m at a depth of 8.17 mm from the cortex surface and 99% of the E-field found in the thalamus was at 13.1 V/m or below. E-fields are shown in Figure 1B.

### Behavioral Tests for Tinnitus

**FIGURE 3:**
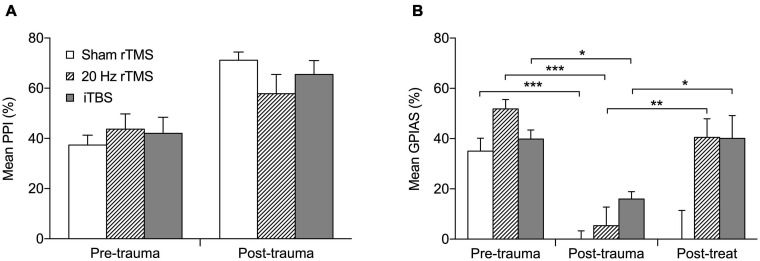
Active rTMS ameliorates behavioral signs of tinnitus. **(A)** The percentage PPI at the frequency of tinnitus development. Mean percentage PPI is significantly different between pre-trauma and post-trauma time points (*p* < 0.0001). **(B)** The percentage GPIAS at the frequency of tinnitus development. For the three animals that developed GPIAS deficit at both 8 and 14 kHz, the average PPI and GPIAS for both frequencies was calculated. Graphs show the data before acoustic trauma (pre-trauma), at the time-point of tinnitus development (post-trauma), and after treatment on the day of final electrophysiological recordings (post-treat; GPIAS data only) for Sham rTMS (*n* = 6), 20 Hz rTMS (*n* = 6), iTBS (*n* = 7). All data mean + SEM, two-way repeated measures ANOVA with Tukey multiple comparisons; *p < 0.05, **p < 0.01, ***p < 0.001.

Before AT all animals showed significant suppression in the PPI test (Figure 3A; Pre-trauma data) as well as in the GPIAS test (Figure 3B; Pre-trauma data), both averaging approximately between 40 and 50%. When tinnitus develops, animals fail GPIAS (a lack of significant suppression between no gap and gap trials within an animal). Although the underlying mechanism of this phenomenon is still under debate (Eggermont, 2013, 2016; Galazyuk and Hébert, 2015; Salloum et al., 2016) the GPIAS test has been verified against psychophysical tests for tinnitus in animals (Turner et al., 2006) and is supported by some data from the human tinnitus population (Fournier and Hébert, 2013, 2020; Duda et al., 2020), although others have debated its use (Campolo et al., 2013; Morse and Vander Werff, 2019). As a lack of significant suppression in the GPIAS test could be due to hearing loss, i.e., the animals not being able to hear the background noise and hence not detecting the gap, or alternatively an issue with startle circuitry, PPI is implemented alongside the GPIAS to exclude these possibilities. Following AT all animals still showed significant suppression of PPI (Figure 3A) indicating that startle circuitry was working normally and that prepulse detection was not significantly affected by ipsilateral hearing loss. Mean PPI suppression was significantly different between the time-points (*F*_2_,_16_ = 0.1733, *p* = 0.8424) and the interaction between treatment and timepoint was non-significant (*F*_2_,_16_ = 1.751, *p* = 0.2052). The increase in PPI following AT may be an indication of hyperacusis being experienced by the animals (Chen et al., 2013; Thomas et al., 2019). Significant PPI suppression indicates that the background noise used in GPIAS should be detectable and a decreased suppression in GPIAS test is not due to their ipsilateral hearing loss. Therefore, a loss of significant suppression in just GPIAS would represent tinnitus.

Nineteen (15 males and 4 females) of the 27 animals developed a GPIAS deficit indicating behavioral signs of tinnitus. The eight non-tinnitus animals consisted of five males and three females and are not further discussed. Tinnitus development occurred between 3 and 16 weeks after AT (7.9 ± 0.8 weeks; mean ± SEM). Twelve of the 19 animals developed a GPIAS deficit at 8 kHz, four animals at 14 kHz, and three animals at both background frequencies. This is in line with our previous studies showing that the GPIAS deficit can occur at either or both frequencies (Mulders et al., 2014a, 2016, 2019; Leggett et al., 2018). The background noise/prepulse center frequencies used for GPIAS and PPI (8 and 14 kHz) were selected as these frequencies are just below the AT frequency in an audiogram region without threshold loss (8 kHz) and in a region just above the AT frequency showing threshold loss (14 kHz). Animals were randomly allocated to a treatment group with the 20 Hz consisting of five males and one female, iTBS groups consisting of six males and one female and the sham group of four males and two females. The average GPIAS suppression in the week of tinnitus development in each group is shown in Figure 3B (post-trauma data), demonstrating the dramatic and significant reduction in suppression (two-way repeated measures ANOVA, *F*_4_,_32_ = 3.33, *p* = 0.0218) as compared to before AT (pre-trauma data). *Post hoc* analysis showed that this significant change was present in all three groups: sham (Tukey’s multiple comparisons test, *p* < 0.001); 20 Hz (Tukey’s multiple comparisons test, *p* < 0.001); iTBS (Tukey’s multiple comparisons test, *p* < 0.05).

After treatment with 20 Hz or iTBS, but not after sham rTMS, GPIAS suppression increased toward pre-AT levels and was significantly higher than at the post-trauma (time of tinnitus development) time-point (Figure 3B; Tukey multiple comparisons; sham, *p* > 0.05; 20 Hz, *p* < 0.001; iTBS, *p* < 0.05). This indicates that both the 20 Hz and iTBS treatment alleviated the signs of tinnitus. GPIAS suppression following sham treatment remained significantly lower than at pre-AT levels, and was unchanged from post-trauma levels, confirming the lack of effect of sham rTMS treatment.

### CAP Audiograms

**FIGURE 4:**
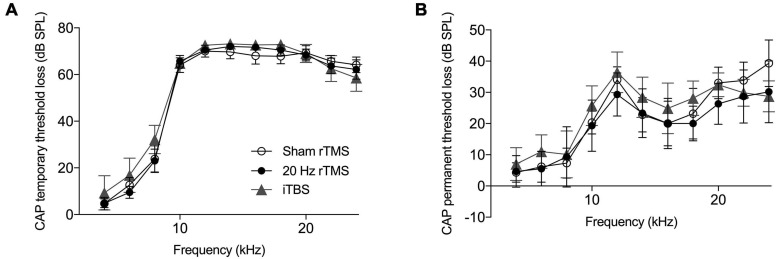
Hearing threshold loss following acoustic trauma. Mean compound action potential threshold loss in the left ear. **(A)** Average CAP threshold loss measured at multiple frequencies (4–24 kHz) immediately following exposure to acoustic trauma. **(B)** Average CAP threshold loss at multiple frequencies (4–24 kHz) at the day of final electrophysiological recordings. All data mean ± SEM. No significant differences between groups identified.

Compound action potential thresholds were measured immediately before and after AT as well as during the final electrophysiological experiment. This was done to ensure that (1) all animals had normal thresholds initially, (2) the AT caused similar effects in all groups, and (3) rTMS and sham treatments did not affect auditory thresholds. The pre-trauma CAP audiogram revealed no pre-existing threshold differences between groups (two-way ANOVA, *F*_20_,_160_ = 0.94, *p* = 0.536) and confirmed normal hearing in all groups (Johnstone et al., 1979). Immediately following AT animals showed a large temporary threshold loss from 6 to 24 kHz, with no significant differences between groups (Figure 4A; two-way ANOVA, *F*_20_,_160_ = 0.45, *p* = 0.980). At the time-point of the final electrophysiological recordings, CAP thresholds in the non-AT ear were normal in all groups (data not shown), but all animals showed a permanent threshold loss in the AT ear (Figure 4B), consistent with our previous guinea pig studies (Mulders and Robertson, 2009, 2011; Mulders et al., 2011). Threshold loss was not significantly different between groups (two-way ANOVA, *F*_20_,_160_ = 0.37, *p* = 0.994), which implies that any differences with regards to tinnitus outcomes, electrophysiology or immunohistochemistry are not due to differences in peripheral thresholds.

### Firing Rates and Patterns in the MGN

**FIGURE 5:**
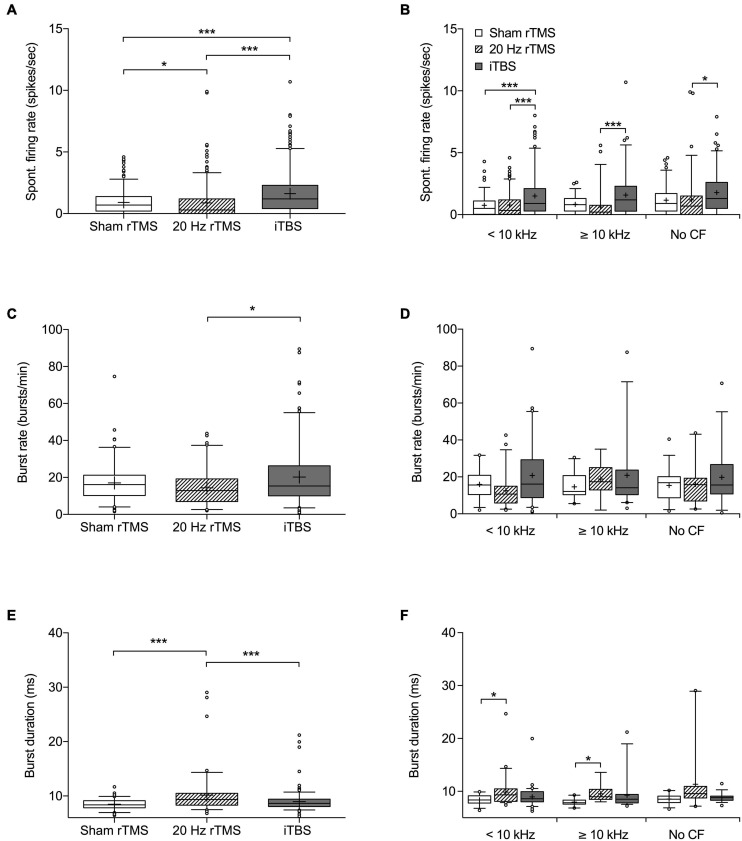
Spontaneous firing rate and burst firing parameters of MGN neurons following rTMS. Box and whisker plot showing **(A)** spontaneous firing rates based on 272 neurons from sham, 295 neurons from 20 Hz rTMS, and 324 neurons from iTBS group. **(B)** Spontaneous firing rate of neurons differentiated into CF groups [< 10 kHz, ≥ 10 kHz, and No CF]. **(C)** Burst firing rate in bursts per minute based on 107 neurons from sham, 86 neurons from 20 Hz rTMS, and 174 neurons from iTBS. **(D)** Burst firing rate of neurons differentiated into CF groups [<10 kHz, ≥10 kHz, and No CF]. **(E)** Burst duration based on 107 neurons from sham, 86 neurons from 20 Hz rTMS, and 174 neurons from iTBS. **(F)** Burst duration of neurons differentiated into CF groups [<10 kHz, ≥10 kHz, and No CF]. All data median with box spanning interquartile range, minimum, maximum and outliers (>95%). Mean is indicated by +. Kruskal–Wallis with Dunn’s multiple comparisons, *p < 0.05, ***p < 0.001.

Spontaneous firing data were collected from a total of 891 neurons (272 from sham rTMS, 295 from 20 Hz rTMS, and 324 from iTBS treated animals) within the right MGN, contralateral to the AT ear, as this represents the main auditory pathway. Spontaneous firing data are non-parametric and are shown as median with associated interquartile range (IQR), there was a significant difference between groups (Figure 5A; Kruskal–Wallis, *p* < 0.0001). MGN neurons from 20 Hz rTMS treated animals had a significantly lower firing rate (0.3 [1.2 IQR] spikes/s) compared to sham rTMS (0.7 [1.2 IQR]; Dunn’s multiple comparisons test, *p* < 0.0286). Whereas, iTBS treated animals had a significantly higher firing rate (1.2 [1.9 IQR] spikes/s) compared to sham rTMS (Dunn’s multiple comparisons test, *p* < 0.0001) and 20 Hz rTMS treated animals (Dunn’s multiple comparisons test, *p* < 0.0001).

Neurons were further analyzed on the basis of CF and pure tone response. Neurons that were pure tone responsive were subdivided into a CF < 10 kHz and ≥10 kHz. This was done as 10 kHz was used as the AT frequency and CAP threshold loss only occurred over and not below the AT frequency. The third group consisted of neurons that responded to noise, but not a pure tone (no CF data). Additionally, differentiated neurons that were just isolated offline based only on wave height and width were excluded from this further analysis. Differentiating neurons in this way indicated a significant difference in spontaneous firing rate between treatment groups (Figure 5B; Kruskal–Wallis, *p* < 0.0001). MGN neurons < 10 kHz from iTBS treated animals had a significantly higher firing rate (0.9[1.8 IQR] spikes/s) compared to sham rTMS (0.5 [1.1 IQR]; Dunn’s multiple comparisons test, *p* < 0.0001) and 20 Hz treated animals (0.35 [1.2 IQR]; Dunn’s multiple comparisons test, *p* = 0.0007). MGN neurons ≥ 10 kHz had a significantly different firing rate between iTBS (1.2 [2.3 IQR] spikes/sec) and 20 Hz rTMS (0.2 [0.75 IQR] Dunn’s multiple comparisons test, *p* < 0.0001), however, active treatment groups were not significantly different from sham treated animals (0.8 [1.0 IQR]; Dunn’s multiple comparisons test, *p* > 0.05). Similarly, the MGN neurons without CF were significantly different between iTBS treated animals (1.3 [2.1 IQR]) and 20 Hz (0.7 [1.5 IQR]; Dunn’s multiple comparisons test, *p* = 0.0018), but not sham treatment (0.9 [4.3 IQR]; Dunn’s multiple comparisons test, *p* > 0.05).

Burst analysis was performed only on neurons with a firing rate > 1 spike/s, resulting in burst analysis on 107 neurons from sham rTMS, 86 neurons from 20 Hz rTMS, and 174 from iTBS treated animals (Figures 5C,E). Burst rates (bursts per minute) were significantly different between the active rTMS treatments (Dunn’s multiple comparisons test, *p* = 0.0119), however there were no significant differences between the active rTMS treatments and sham rTMS (Dunn’s multiple comparisons test, *p* > 0.05) (Figure 5C; Kruskal–Wallis, *p* = 0.0150). Differentiating MGN neurons on the basis of CF (<10 kHz, ≥ 10 kHz or no CF) indicated no significant differences in burst firing rate between treatment groups (Figure 5D; Kruskal–Wallis, *p* > 0.05).

Mean burst duration was slightly but significantly longer after 20 Hz rTMS treatment (9.36 [2.19 IQR] ms), compared to sham rTMS (8.38 [1.29 IQR] ms; Dunn’s multiple comparisons test, *p* < 0.0001) and iTBS treatments (8.65 [1.36 IQR] ms; Dunn’s multiple comparisons test, *p* = 0.0006) (Figure 5E; Kruskal–Wallis, *p* < 0.0001). Differentiating neurons based on CF (<10 kHz, ≥10 kHz or no CF) indicated significant differences in burst duration between treatment groups (Figure 5F; Kruskal–Wallis, *p* < 0.0001). MGN neurons < 10 kHz from 20 Hz rTMS treated animals had a significantly longer burst duration (9.34 [2.35 IQR]) compared to sham rTMS (8.38 [1.137 IQR]; Dunn’s multiple comparisons test, *p* = 0.0315), but not iTBS treated animals (8.61 [1.9 IQR]). Similarly, MGN neurons ≥ 10 kHz from 20 Hz rTMS treated animals were significantly longer (8.98 [1.88 IQR]) than sham rTMS (7.86 [0.78 IQR]), but not iTBS treated animals (8.52 [1.62 IQR]). MGN neurons that responded to noise, but not a pure tone (no CF data) were not significantly different between treatment groups.

### Immunohistochemistry

**FIGURE 6:**
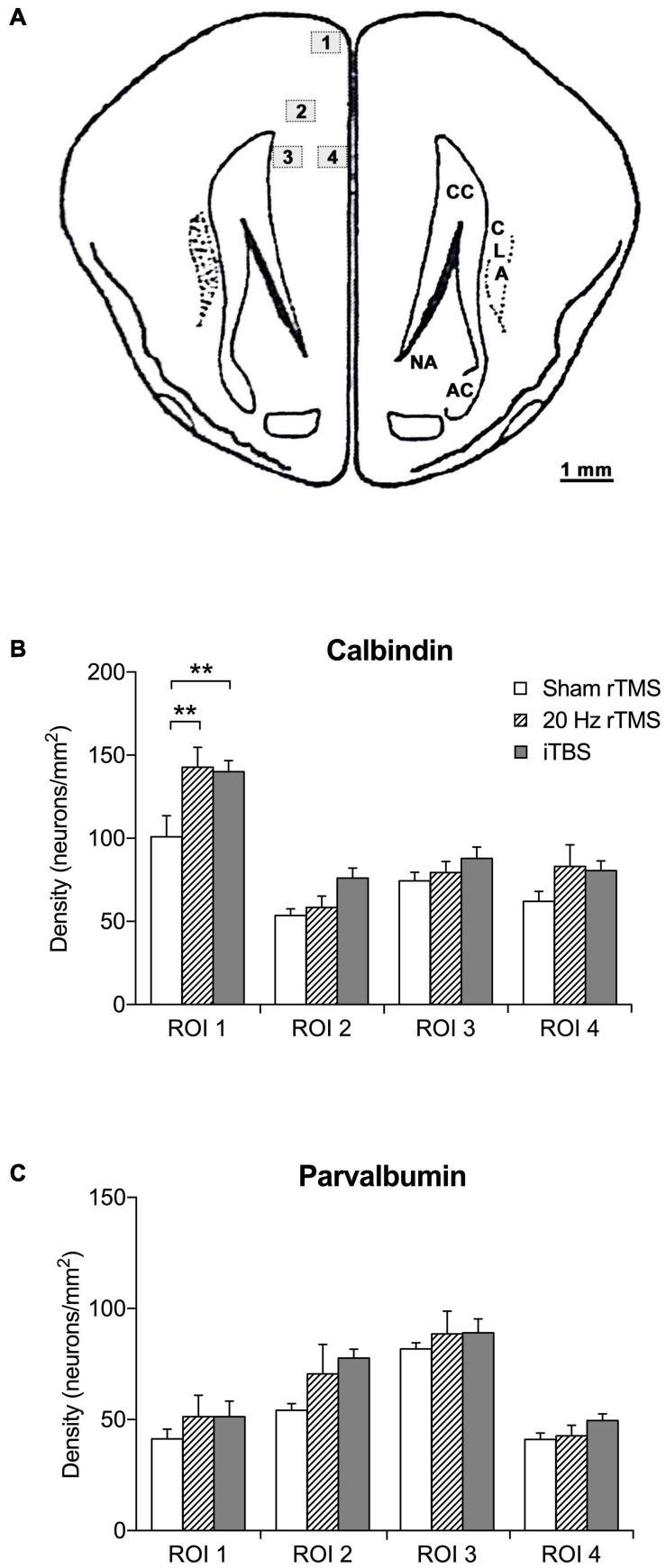
Regions of interest and density of calbindin- and parvalbumin-positive neurons in the PFC. **(A)** Schematic illustration of location and area size of regions of interest (ROI). The counting frame area was set to 0.24 mm^2^ using a X20 objective lens. The mean density per mm^2^ was calculated by the mean number of calcium-binding immunoreactive neurons divided by the frame area (mm). Numbered boxed (1–4) represent locations and area (mm^2^) of ROI. Image adapted from Rapisarda and Bacchelli (1977). **(B)** Mean density of calbindin-positive neurons in sham (*n* = 6), 20 Hz rTMS (*n* = 6), and iTBS (*n* = 7) treated animals within four ROI in the PFC. **(C)** Mean density of parvalbumin-positive neurons in sham (*n* = 6), 20 Hz rTMS (*n* = 6), and iTBS (*n* = 7) treated animals within four ROI in the PFC. All data mean + SEM, repeated measures ANOVA with Sidak’s multiple comparisons; **p < 0.01).

TMS treatments have been shown to alter the densities of calcium-binding proteins in the cortex (Benali et al., 2011; Volz et al., 2013; Castillo−Padilla and Funke, 2016; Makowiecki et al., 2018; Mulders et al., 2019). In agreement, the densities of calbindin-positive neurons were significantly different between groups (two-way repeated measures ANOVA, *F*_6_,_48_ = 2.5, *p* = 0.0360). This significant increased density compared to sham treatment was present in ROI 1 (the most dorsal ROI, see Figure 6A) after both the 20 Hz rTMS (Sidak’s multiple comparisons, *p* = 0.0020) and iTBS treatment (Sidak’s multiple comparisons, *p* = 0.0027), but the other ROIs did not show a statistically significant difference (Figure 6B). The densities of parvalbumin-positive neurons were not significantly different (Figure 6C; two-way repeated measures ANOVA, *F*_6_,_48_ = 1.1, *p* = 0.4008).

## Discussion

The present study examined the effects of two excitatory rTMS protocols, commonly used in clinical settings, applied over PFC in an animal model of tinnitus. Both rTMS treatments resulted in a significant decrease in the behavioral signs of tinnitus compared to sham treatment. An increase in the density of calbindin within the most dorsal region of the PFC confirmed local effects of rTMS on the targeted area. Interestingly, the attenuation of tinnitus was accompanied by significant changes in spontaneous neuronal firing rate in opposite directions following the active rTMS protocols. Burst rate in the MGN was significantly different between 20 Hz rTMS and iTBS treatment, but there was only a significant change in burst duration compared to sham following 20 Hz rTMS. Collectively, the present findings provide support for the use of rTMS of the PFC to ameliorate tinnitus in humans, and lend support to current hypotheses implicating the PFC in the generation of tinnitus (Rauschecker et al., 2010, 2015). However, our data also suggest that the therapeutic benefit of stimulating PFC may not always be directly associated with inhibition of thalamic hyperactivity.

A key element of our experimental design was that the thalamus should not be directly stimulated by rTMS, but rather be indirectly activated by stimulation of the PFC. A study of the action potential thresholds of pyramidal cells in cortical slice preparations using stimulating electrodes suggested that ∼28 V/m was the minimum E-field intensity that could produce an action potential in some pyramidal neurons (Radman et al., 2009), although mean value was 57 ± 6 mV/mm for layer V/VI neurons and 81 ± 3 mV/mm for layer II/III pyramidal neurons. In addition, findings from computational models suggest that GABAergic interneurons have higher input resistance compared to pyramidal neurons (Hausser et al., 2000; Markram et al., 2004), and therefore may be even less susceptible to direct depolarization by the induced E-fields of TMS (Aberra et al., 2020). Modeling of the intensity of the E-field induced by our stimulation protocol confirmed that the thalamus received intensities well below the threshold for action potentials (∼20 V/m), while the superficial layers of the cortex were stimulated at intensities within the lower range shown to elicit action potentials (up to 39.5 V/m). The E-field intensities at the 23% machine stimulus output used in this experiment are also below the motor threshold of 28% machine stimulus output using the same Cool-40 coil (Parthoens et al., 2016), determined using direct measurements of motor evoked potentials. Thus, our stimulation protocol likely induced action potentials in some pyramidal neurons, mostly in the superficial layers of the cortex, but did not directly activate thalamic neurons. However, E-field values below the threshold value required to produce action potentials have been observed inducing non-synaptic mechanisms of neuroplasticity such as structural reoganisation (Makowiecki et al., 2014), increased neurogenesis (Heath et al., 2018), gene regulation and increased intracellular calcium (Grehl et al., 2015; Clarke et al., 2017b), altered neuron excitability (Tang et al., 2016; Makowiecki et al., 2018), and glial changes (Clarke et al., 2017a; Cullen et al., 2019). Therefore, we cannot rule out that non-synaptic mechanisms of plasticity induced in the thalamus by low intensity magnetic fields may contribute to the changes in firing parameters that we report here.

The positive effects on tinnitus following both treatments, are in agreement with multiple clinical studies, which have reported beneficial effects on tinnitus following combined rTMS treatment over PFC and auditory cortex (Kleinjung et al., 2008; Lehner et al., 2013; Langguth et al., 2014; Ciminelli et al., 2020), most likely due to resulting inhibitory effects on auditory cortex. The mechanisms by which activation of PFC leads to attenuation of tinnitus remain to be elucidated. One possible mechanism would be that the observed amelioration of tinnitus is due to effects of PFC on the MGN and consequently on auditory cortex. Prior research has indicated direct and indirect projections from PFC to the thalamic reticular nucleus (Cornwall et al., 1990; Uylings and van Eden, 1991; Zikopoulos and Barbas, 2006) and the latter, in turn, has primarily inhibitory inputs to MGN (Yu et al., 2009). Hence, excitatory effects from our rTMS protocols on PFC (Pascual-Leone et al., 1994; Maeda et al., 2000; Huang et al., 2005; Pell et al., 2011) are thought to lead to activation in the thalamic reticular nucleus, which would result in inhibitory effects in MGN, which in turn would lead to inhibition of cortical activity.

However, despite the similar behavioral outcomes, with both active protocols ameliorating tinnitus, the two excitatory protocols affected activity in MGN differently: following 20 Hz rTMS, animals exhibited a decrease in spontaneous firing rate, no change in burst rate and a small yet significant increase in burst duration, whereas iTBS animals exhibited an increase in firing rate (both tonic and burst firing). The reason for these divergent effects after two excitatory protocols remains to be elucidated. The possibility exists that we recorded from different subdivisions in the MGN, however it is unlikely that there would be a consistent bias toward one subdivision in one group of animals and not in the other groups. It also seems unlikely that it is linked to the use of anesthesia, although our anesthesia protocol is likely to have resulted in lower overall spontaneous activity as we have discussed previously using our animal model (Cook et al., 2021).

The decreased activity that was observed in MGN after 20 Hz stimulation would be in line with the circuitry outlined above. However, the increased activity in the MGN following iTBS was unexpected: first, in view of the pathway outlined above and second, because an increase in firing rates in the auditory pathway is associated with the presence of tinnitus (Kalappa et al., 2014; Wu et al., 2016). Indeed, we have shown in our animal model that there is an increased tonic spontaneous firing in the MGN of animals with tinnitus compared to animals without tinnitus (Cook et al., 2021) and hence we expected reduced spontaneous firing rates in the MGN following both active protocols alongside the attenuation of tinnitus.

One possibility to explain how the different rTMS protocols caused similar behavioral outcomes despite different changes in MGN activity is that stimulation of PFC ameliorated the behavioral signs of tinnitus after iTBS treatment independently of MGN, possibly via a direct pathway linking PFC and auditory cortex (Pandya and Kuypers, 1969; Petrides and Pandya, 1988; Winkowski et al., 2018). Indeed, human studies have shown that tinnitus is associated with a dysfunctional pathway from PFC to auditory cortex (Song et al., 2015) and it may be this pathway that is responsible for the therapeutic effects observed. Further studies measuring activity in auditory cortex rather than MGN, after PFC rTMS stimulation are required to investigate this possibility.

Alternatively, the changes in burst firing induced by the rTMS protocols may have an impact on tinnitus perception and maintenance. Thalamic burst firing has been postulated to play a role in salience in alert states and may be involved in pathological conditions such as phantom perceptions (Llinás et al., 1999; Sherman, 2001; Llinás and Steriade, 2006). However, contrasting theories exist regarding the role of burst firing. The thalamocortical dysrhythmia theory proposes that in cases of phantom perception, such as tinnitus, disfacilitation or increased inhibition in the MGN, produces low-threshold calcium bursts, causing abnormal oscillations between the thalamus and cortex (Llinás et al., 1999; De Ridder et al., 2015). In the cortex, this abnormal oscillation pattern is detectable as gamma oscillations, which can produce a conscious sensory percept (Llinás et al., 1999; van der Loo et al., 2009; Caspary and Llano, 2017). This theory is supported by the results of Kalappa et al. (2014) who showed increased spontaneous burst firing in animals with tinnitus after an AT compared to unexposed controls without tinnitus. However, interestingly, data arising from the research area of chronic pain show that increased burst firing has been associated with inhibition of pain (Kim et al., 2003; Cheong et al., 2008). Indeed, a recent study showed that inducing thalamic bursts, using optogenetic activation of the thalamic reticular nucleus neurons, resulted in down-regulation of nociceptive behavior in awake mice (LeBlanc et al., 2017). Therefore, the significantly higher burst firing (increased burst rate in iTBS and increased burst duration in 20 Hz rTMS) observed following rTMS treatment in the present study could potentially serve to prevent the transmission of the tinnitus signal to the cortex.

In addition, high frequency rTMS of the PFC has been shown to affect the neurochemical make-up of the mesolimbic system, modulating dopamine in the anterior cingulate gyrus in healthy humans (Cho and Strafella, 2009) and in the dorsal hippocampus, the shell of the nucleus accumbens and the dorsal striatum in rats (Keck et al., 2000). Structural and functional malfunction of the mesolimbic system has been implicated in depression and chronic pain (Belujon and Grace, 2017; Serafini et al., 2020) and in addition, the circuitry thought to be involved in chronic pain and tinnitus shows remarkable overlap (Rauschecker et al., 2015). Hence, alteration of the mesolimbic system by rTMS stimulation may also be involved in the positive effects observed on tinnitus perception.

Even though the two rTMS protocols influenced thalamic activity in different ways, the effects on the density of the calcium-binding protein calbindin in the PFC were similar which is in line with both protocols being excitatory. Calbindin densities were increased in the most dorsal layers of the PFC only, which suggest a proximity-dependent relationship between density and the rTMS coil. The increase in calbindin neuron densities may be due to a reduction of protein degradation or increased protein synthesis. The increased density is in agreement with our previous study applying low-intensity rTMS stimulation to PFC (Mulders et al., 2019) and another study using rTMS over mouse visual cortex (Makowiecki et al., 2018). However, a study using 2 weeks of iTBS in rats showed reduced parvalbumin expression (Castillo−Padilla and Funke, 2016). This discrepancy may be due to species differences (Lensjø et al., 2017) or differences in baseline cortical activity (Makowiecki et al., 2018). How the increased density is related to PFC activity remains to be investigated. Parvalbumin and calbindin are both present in inhibitory neurons in the cortex (Druga, 2009; Atallah et al., 2012). In view of the excitatory protocols used (Pascual-Leone et al., 1994; Maeda et al., 2000; Huang et al., 2005; Pell et al., 2011), the higher density of immunopositive neurons could be the result of a homeostatic response to increased glutamatergic neuronal activity.

Finally, our data also suggest that the animals may have developed hyperacusis after the AT as the PPI showed a significant increase following AT, which has been suggested as a marker for hyperacusis (Chen et al., 2013; Thomas et al., 2019). Hyperacusis is a common co-morbidity in tinnitus patients (Schecklmann et al., 2014), as both are strongly associated with hearing loss (Baguley et al., 2013; Paulin et al., 2016). Recent studies in human subjects with tinnitus with or without hyperacusis suggest that hyperacusis is associated with increased neural gain which shown as an increase in sound evoked activity whereas tinnitus is associated with increased noise and reduced activity at the tinnitus frequency (Hofmeier et al., 2021; Koops and van Dijk, 2021). Unfortunately, in the present study, PPI was not measured after the rTMS treatments, which would be a useful additional measure in future studies to provide further information regarding the neural substrates underlying tinnitus and hyperacusis.

In summary, our study indicates that excitatory rTMS over PFC results in significant improvements in tinnitus. The mechanism behind this improvement needs further study to elucidate the exact circuitry.

## Data Availability Statement

The raw data supporting the conclusion of this article will be made available by the authors, without undue reservation.

## Ethics Statement

The animal study was reviewed and approved by Animal Ethics Committee of The University of Western Australia.

## Author Contributions

WM and JR contributed to conception and design of the study. JZ and HT collected the data and performed the statistical analysis. KB contributed to data processing of electrophysiological single neuron recordings. KL contributed to data collection. JZ wrote the first draft of the manuscript. WM, JR, and SB wrote sections of the manuscript. SB produced guinea pig brain models and simulated induced electric fields. All the authors contributed to manuscript revision, read, and approved the submitted version.

## Conflict of Interest

The authors declare that the research was conducted in the absence of any commercial or financial relationships that could be construed as a potential conflict of interest.
